# Child’s Play: Examining the Association Between Time Spent Playing and Child Mental Health

**DOI:** 10.1007/s10578-022-01363-2

**Published:** 2022-05-14

**Authors:** Helen F. Dodd, Rachel J. Nesbit, Lily FitzGibbon

**Affiliations:** 1 Children and Young People’s Mental Health Research Collaboration (ChyMe), College of Medicine and Health, University of Exeter, 2.05b South Cloisters, St Luke’s Campus, Heavitree Road, Exeter, EX1 2LU UK; 2 School of Psychology and Clinical Language Sciences (PCLS), University of Reading, Reading, UK

**Keywords:** Play, Child mental health, Anxiety, Internalising problems

## Abstract

It is theorised that adventurous play offers learning opportunities that help to prevent mental health problems in children. In this study, data from two samples is used to examine associations between the time that children aged 5–11 years spent playing adventurously and their mental health. For comparison, time spent playing unadventurously and time spent playing outdoors are also examined. Study 1 includes a sample of 417 parents, Study 2 includes data from a nationally representative sample of 1919 parents. Small, significant associations between adventurous play and internalising problems, as well as positive affect during the first UK-wide Covid-19 lockdown, were found; children who spend more time playing adventurously had fewer internalising problems and more positive affect during the Covid-19 lockdown. Study 2 showed that these associations were stronger for children from lower income families than for children from higher income families. The results align with theoretical hypotheses about adventurous play.

**Supplementary Information:**

The online version contains supplementary material available at 10.1007/s10578-022-01363-2.

## Introduction

Recent research estimates that, outside of school, children in Britain spend just over three hours a day playing [1]. Play is intrinsically rewarding for children, provides a way for them to interact with the world, express themselves, and make sense of the world around them. Despite this, there has been growing concern that children’s play, particularly their outdoor, independent, adventurous play has been declining over recent decades. For example, Clements [2] found that whilst 70% of mothers surveyed in the United States reported that they played outdoors daily as children, only 31% of their children were reported to do so. Moreover, findings from the UK suggest that whilst parents reported spending approximately 40% of their time playing as children in natural spaces, only 10% of their children’s play was reported to happen in these spaces [3]. Furthermore, there is robust evidence of increasing restrictions over the past 50 years on children’s independent mobility, defined as the age at which children are allowed out alone and how far they allowed to travel [1, 4]. Alongside these declines in outdoor play and independence, rates of emotional disorders in UK children aged 5–16 years increased by 49% between 1994 and 2017 [5] and recent data show further increases, with an estimated one in six UK children having a probable mental health problem in July 2020 [6]. It has been proposed that reductions in children’s outdoor, risky play, may have negative consequences for children’s mental health, and may be contributing in part to the increase in children’s mental health problems [7–9]. To date though, no empirical research has directly examined this association [10].

Adventurous play, or risky play, is defined as child-led play where children experience subjective feelings of excitement, thrill and fear; often in the context of age-appropriate risk-taking. Several theoretical articles have hypothesised links between adventurous play and children’s mental health, particularly internalising problems such as anxiety and phobias. For example, Sandseter and Kennair [9] propose that children have a natural drive to engage in risky play, which evolved because it has anti-phobic effects, naturally exposing children to stimuli that may otherwise be feared such as heights and water. In keeping with this, Gray [7] theorises that the decline in play over the last generation is associated with increasing rates of mental health problems in children. A recent conceptual model drew on the extensive literature related to the development of anxiety in children to argue that sufficient adventurous play experiences during childhood may help to prevent anxiety in children [8]. Specifically, Dodd and Lester describe the role of intolerance of uncertainty [11], coping [12, 13], anxiety sensitivity [14] and avoidance [15, 16] in child anxiety and propose that adventurous play provides a motivating, positive context for exposures to, and subsequent healthy learning about, uncertainty, coping and physiological arousal. They argue that through these learning experiences children’s ability to cope adaptively in the face of uncertainty and arousal increases and their risk for problematic anxiety decreases.

Despite this theoretical work, research examining the association between adventurous play and children’s anxiety, or mental health more broadly defined, is scarce. Qualitative evaluations of school-based interventions to increase levels of adventurous play report positive outcomes of adventurous play, including increases in resilience [17] and improvements in children’s happiness at school [18]. There is also evidence that play, in general, is good for children’s wellbeing and mental health; children admitted to hospital show lower levels of anxiety and fewer negative emotions when they take part in a play intervention [19] and, when given time for free play, hospitalised children show reductions in stress [20]. Outdoor play and being in nature, which facilitate adventurous play, also have a positive effect on children’s wellbeing [21–23], but the direct link between adventurous play and children’s anxiety has yet to be examined.

Although the theoretical links between play and internalising symptoms have been described in previous work, to our knowledge adventurous play has not been explicitly linked to externalising problems such as Attention Deficit Hyperactivity Disorder (ADHD) and Conduct Disorder within psychological theory. There are some findings that would be consistent with adventurous play offering benefits for externalising problems. For example, schools taking part in an intervention to prioritise risk and challenge in the playground reported less bullying and conflicts on the playground post-intervention [18]. Further, children with ADHD who played regularly in green play settings were reported to have milder symptoms than children who played in built outdoor or indoor play settings [24]. It is not clear though that adventurous play has a specific role in these positive effects and, again, there is a lack of research directly evaluating the link.

In this paper we present two studies which examine the association between parent-report of children’s time spent playing and child mental health. Study 1 and Study 2 use the same measures of play and mental health; Study 1 uses an opportunity sample of parents living in Northern Ireland and Study 2 examines the extent to which the findings hold using a large nationally representative sample of parents living in Great Britain. To capture mental health broadly, in both studies we evaluate long term symptoms of internalizing and externalizing problems as well as shorter term positive and negative affect, which were assessed during the first weeks of the Covid-19 lockdown. Internalising problems include symptoms of anxiety and depression whereas externalising problems refer to difficulties with behaviour and attention. Positive and negative affect refer to more transient mood states. Time spent playing adventurously, time spent playing unadventurously and time spent playing outdoors are estimated using the Children’s Play Scale (CPS; 25) in both studies.

Our primary hypothesis follows relevant conceptual models: that children who spend more time playing adventurously will have fewer internalizing symptoms. In addition, we hypothesise that children who typically spend more time playing adventurously will have less negative affect and more positive affect during the Covid-19 lockdown, which we conceptualise as indicating better coping under uncertainty. To evaluate the specificity of these associations, we also examine associations between adventurous play and externalizing symptoms, as well as associations between mental health and both time spent playing unadventurously and time spent playing outdoors.

## Study 1

### Method

#### Participants

Survey respondents were 427 parents/caregivers (93% female) with a child aged 5 to 11 years. Participants were self-selecting, recruited via social media and newsletters sent from a range of organizations. Their children (45% female) were aged 5 to 11 years (M = 8.02, SD = 1.98). All participants were living in Northern Ireland and the majority identified as White British or White Irish, consistent with the population of Northern Ireland, where > 98% are white. There was diversity in other demographic factors, see Table S1 in Supplementary Material for full demographic characteristics. To be included, participants had to correctly answer two questions that were designed to screen out bots and participants not paying attention (see Section 2 of Supplementary Material for details).

#### Measures

The full survey was designed to mirror the British Children’s Play Survey (BCPS; 1), which did not include participants in Northern Ireland. The focus in the present paper is on associations between children’s play and mental health. For completeness we analysed the data addressing the same research questions as the published BCPS paper and present these results in Supplementary Material Section 4.

##### Children’s Play Scale (CPS)

The CPS [25] includes questions about play in seven places. Full details are provided in Supplementary Materials Section3. Briefly, respondents report the frequency and length of time their child plays in each place in Spring/Summer and Autumn/Winter. These responses are then used to give an estimate of total time spent playing in each place within a year. The adventurous play supplement asked parents to rate how adventurously their child plays at each place. This was rated on a five-point Likert scale ranging from 1 (very low levels of adventure) to 5 (maximum levels of adventure). For this study we extracted three variables: total time spent playing outdoors, time spent playing adventurously and time spent playing unadventurously. To calculate time spent playing adventurously, we calculated the total time spent playing using only places where parents rated that their child played with at least a mild level of adventure (2 on the Likert scale). To calculate time spent playing unadventurously, we calculated the total time spent playing using only places where parents rated that their child played with very low level of adventure (1 on the Likert scale). We conducted sensitivity analyses with the cut off at 2 for unadventurous and 3 for adventurous and the pattern of results and conclusions remained the same. The test re-test and cross-informant reliability of the CPS and adventurous play supplement have been evaluated in Dodd, Nesbit [25].

##### SDQ

The SDQ is a 25-item screening questionnaire that asks about positive and negative attributes. Items can be combined to create five subscales: emotional symptoms, conduct problems, hyperactivity/inattention, peer relationship problems and prosocial behaviour. An internalising problems score can be calculated by summing emotional problems and peer relationship problems, and an externalising score can be calculated by summing conduct problems and hyperactivity/inattention. The SDQ is widely used and has strong psychometrics (see www.sdqinfo.org [26]).

##### PANAS

The 10-item Positive and Negative Affect Schedule for Children-P [27] asks parents to read each of 10 items which describe an emotion and select to what extent their child has felt this way during the past few weeks. Five items are positive emotions, five are negative. Responses are summed to give scores for positive affect and negative affect. The scale has good psychometric properties [27].

##### Kessler-6 (K6)

The Kessler-6 [28] is a 6-item scale, designed to capture distress, based on symptoms of anxiety and depression, in adults. The original scale asks respondents to consider the previous 4-week period and to rate, using a five-point Likert scale, how often they felt each of the six emotions listed. To gain a measure of longer-term parent mental health, which we use as a control variable, we asked respondents to describe how often they had felt each emotion over the past year. The K6 has good psychometric properties [29].

#### Procedure

Participants were recruited via social media and newsletters sent from a range of organizations. Interested parents were invited to take part in the study via advertisements that directed them to an information sheet and consent form. After consent the survey opened in SurveyMonkey. Recruitment began on 3rd April 2020, when the first responses to the survey were received. The survey was closed on 26th April 2020. The UK-wide lockdown due to Covid-19 began on March 26th meaning the survey was always completed within one month of the start of the lockdown. Respondents completed the survey on one occasion and were clearly instructed to answer the CPS and SDQ questions thinking about life before it was affected by the Covid-19 pandemic. For the PANAS they were asked to respond with the previous few weeks in mind. Participants were given a £10 voucher.

#### Missing Data, Distributions and Outlier Checks

There was some missing data, in particular on three of the time questions on the CPS. Missing data were imputed using the mice package in R [30] and pooled results are reported. Where variables were skewed they were transformed and outliers were Winsorized. See supplementary materials Sect. 2 for full details.

#### Data Analysis Plan

Given that analyses included three variables extracted from the CPS we examined correlations between these measures. Time spent playing adventurously was positively associated with time spent playing outside (*r* = 0.59, *p* < 0.001) and negatively associated with unadventurous play (*r* =  − 0.63, *p* < 0.001). Time spent playing outside and unadventurous play were not significantly correlated (*r* = 0.07, *p* = 0.168).

To examine our hypotheses, Pearson *r* correlations, pooled from the multiply imputed datasets, were used to evaluate associations between the three play variables and four child mental health variables (SDQ internalising, SDQ externalising, PANAS positive affect, PANAS negative affect). A Bonferroni-corrected alpha of 0.0125 (0.05/4) was used to correct for multiple comparisons. We then examined whether these correlations remained significant after controlling for parent mental health and child sex, child age, child disability or mental health problem, child birth-order, parent employment status, parent marital status, parent age and parent level of education. Again, a Bonferroni corrected alpha level of 0.0125 was used.

### Results

All correlations can be seen in Table 1. These results show that time spent playing adventurously was significantly associated with both children’s internalising scores on the SDQ and positive affect as measured using the PANAS, with children who spent more time playing adventurously having fewer internalising symptoms and more positive affect. No significant correlations were found between adventurous play and either externalising symptoms or negative affect. In contrast, hours spent playing unadventurously was not related to positive affect and was positively related to internalising scores on the SDQ. Children who spent more time playing unadventurously had more internalising symptoms. For hours spent playing outdoors, no significant correlations with internalising or externalising scales nor for PANAS positive or negative affect scales were found.

**Table 1:** Mean and standard deviation for each child mental health measure and Pooled Pearson correlation coefficients (r) and 95% confidence intervals for relationships between play variables and children's mental health

	PANAS negative affect	PANAS positive affect	SDQ externalising	SDQ internalising
Mean (standard deviation)	8.87 (3.31)	17.52 (3.41)	5.51 (3.81)	4.20 (3.51)
Hours playing adventurously (per year)	− 0.02 [− 0.11, 0.08]	0.12* [0.03, 0.22]	− 0.02 [− 0.11, 0.08]	− 0.19* [− 0.28, − 0.10]
Hours playing unadventurously (per year)	− 0.05 [− 0.14, 0.05]	− 0.02 [− 0.12, 0.07]	− 0.05 [− 0.15, 0.04]	0.12* [0.03, 0.22]
Hours playing outside (per year)	0.02 [− 0.08, 0.11]	0.08 [− 0.02, 0.18]	− 0.05 [− 0.14, 0.05]	− 0.09 [− 0.19, 0.00]

*p < 0.0125

All three significant correlations were robust to controlling for demographic variables. The full results of these regressions can be seen in the results file here: https://beta.ukdataservice.ac.uk/datacatalogue/doi/?id=8793#!#0. Although it was not our primary focus, in addition to the play variables, the following demographic factors were significant predictors of child mental health. For both regressions predicting internalising problems (one with adventurous play as a predictor and one with unadventurous play as a predictor), the demographic variables that were significant predictors were child disability status, marital status and parent mental health (*p* < 0.05). Children had fewer internalising symptoms when they were not described as having a learning disability, mental health problem or physical disability, when their parents were married or living as married and when the reporting parent had lower levels of distress as reported on the K6. For the model predicting positive affect, the demographic variables that were significant predictors were child sex, child age, child disability status and parent mental health (*p* < 0.05). Children had more positive affect if they were female, younger, did not have a disability, and if their parent reported lower distress on the K6.

### Brief Discussion

It was hypothesised that children who spend more time playing adventurously would have fewer internalizing symptoms and that children who typically spent more time playing adventurously would have less negative affect and more positive affect during the first weeks of the Covid-19 lockdown. Results support these hypotheses with the exception that there was no evidence of an association between adventurous play and negative affect.

The results provide some indication of specificity because no significant association was found between adventurous play and externalising problems. Furthermore, there was a positive association between time spent playing unadventurously and internalising problems, and no significant correlation between mental health and time spent playing outdoors, which further suggests specificity.

A limitation of this research is that the sample, although relatively diverse, was not recruited to be representative of the general population. Given the small effect sizes and confidence intervals close to zero, replication of these findings is important.

## Study 2

The aim of Study 2 was to further evaluate the hypotheses outlined within Study 1 via analysis of data from the British Children’s Play Survey.

### Methods

#### Participants

Participants were 1919 respondents who took part in the British Children’s Play Survey, administered by YouGov, a UK public opinion research company. The sample were recruited to be approximately nationally representative and then weighted back to the national profile of all adults aged 18 + including those without internet access. All participants (54% female) were parents or caregivers of children (49% female) aged 5 to 11 years. Full details of the sample are provided in Dodd et al. [1].

#### Measures

The measures were identical to those detailed in Study 1, including the CPS, SDQ, PANAS and K-6.

#### Procedure

Participants were invited to take part via email and directed to an online survey which they completed once. Recruitment started on 4th April 2020 and data were collected 4th-15th April 2020. The UK-wide lockdown due to Covid-19 began on 26th March 2020 so all data were collected within 3 weeks of the start of lockdown. As in Study 1, respondents were given very clear instructions to answer the questions thinking about life before it changed as a result of the pandemic, with the exception of the PANAS, where they were asked to respond with the previous week in mind.

#### Missing Data, Distributions and Outlier Checks

There were some missing data, in particular for a single item on the PANAS. Missing data was imputed using the mice package in R [30] and pooled results are reported. Where variables were skewed they were transformed and outliers were Winsorized. See Supplementary Materials Section 3 for details.

#### Data Analysis Plan

Following Study 1, pooled Pearson correlations were used to evaluate associations between the play variables and four child mental health variables. A Bonferroni corrected alpha of 0.0125 (0.05/4) was used. Additionally, in keeping with Study 1, we also examined whether the results held after controlling for parent mental health and demographic variables. Linear regressions were conducted to examine whether associations remained significant after including child age, child sex, child disability, parent disability, employment status, birth order, parent age, parent education and parent mental health as predictors.

### Results

The correlations are shown in Table 2. These results show that effect sizes of all associations were small. Nevertheless, hours spent playing adventurously was significantly associated with both children’s internalising scores on the SDQ and positive affect as measured using the PANAS, with children who spent more time playing adventurously having fewer internalising symptoms and more positive affect. In contrast, hours spent playing unadventurously was not related to positive affect nor to SDQ scores. Children who spent more time playing unadventurously had less negative affect. For total time spent playing outdoors, significant correlations were found with both SDQ internalising scores and PANAS positive affect scores. No significant correlations were found between any of the time spent playing variables and externalising scores. All of the significant associations were robust to controlling for demographic variables in a linear regression (see results file available: https://beta.ukdataservice.ac.uk/datacatalogue/doi/?id=8793#!#0).

**Table 2:** Mean and standard deviation for each child mental health measure and Pooled Pearson correlation coefficients (r) and 95% confidence intervals for relationships between play variables and children's mental health

	PANAS negative affect	PANAS positive affect	SDQ externalising	SDQ internalising
Mean (standard deviation)	10.09 (3.96)	16.14 (4.02)	6.56 (3.83)	4.75 (3.69)
Hours playing adventurously	0.04 [− 0.01, 0.09]	0.18* [0.13, 0.22]	0.04 [− 0.01, 0.08]	− 0.06* [− 0.11, − 0.02]
Hours playing unadventurously	− 0.09* [− 0.13, − 0.04]	− 0.05 [− 0.10, − 0.01]	− 0.03 [− 0.07, 0.02]	− 0.01 [− 0.06, 0.03]
Hours playing outside	0.07 [0.01, 0.12]	0.12* [0.07, 0.16]	0.02 [− 0.02, 0.07]	− 0.07* [− 0.11, − 0.02]

*p < 0.0125

Although it was not our primary focus, in addition to the play variables, the following demographic factors were significant predictors of child mental health. For the regressions predicting SDQ internalizing scores, child disability, parent disability, birth order, parent age and parent mental health were all significant predictors (*p* < 0.05); children had fewer internalizing symptoms if they did not have a learning disability, physical disability or diagnosed mental health problem, were not first born, had parents who were older and parents who were experiencing less distress as measured using the K6. For regressions predicting positive affect, child age, parent education and parent mental health were significant predictors (*p* < 0.05), with children having more positive affect when they were younger, female, and when their reporting parent had lower distress as reported on the K6. For regressions predicting negative affect, child sex, child disability, parent disability, parent employment status, parent age, parent education and parent mental health were all significant predictors (*p* < 0.05). Children had less negative affect when they were male, did not have a disability, did not have a parent with a disability, when their parents were not working, older, highly educated and experiencing less distress as measured on the K6.

The results quite closely replicate the findings from Study 1 with the exception that the correlation between adventurous play and internalising problems was notably smaller. Given that the Study 1 sample were from Northern Ireland, where human capital per head and household income are lower than across Scotland, Wales and England [31, 32], we speculated that the difference in results might be explained by demographic differences between the samples. The BCPS dataset included a binary variable labelled ‘Social Grade’. This is a classification based on the occupation of the lead income earner in the household and is closely associated with household income [33]. In the absence of household income data we chose to use this variable to explore whether results would differ between children growing up in higher income and lower income households.

Household income and play were entered as predictors in linear regression models along with their interaction. Separate regressions were conducted for each of the mental health measures and each play variable. A Bonferroni corrected alpha of 0.0125 was used given the four mental health measures. Full models are available in the results file available here: https://bit.ly/3lqxXdx. Significant interactions were found for five models. Two of these models were for SDQ internalising score; one with a significant interaction between adventurous play and household income (*b* =  − 0.01 [− 0.02, 0.00], *p* = 0.002) and the second with a significant interaction between outdoor play and household income (*b* =  − 0.01 [− 0.02, 0.00], *p* = 0.004). In one model for positive affect, a significant interaction between adventurous play and household income was found (*b* = 0.04 [0.01, 0.06], *p* = 0.003). Finally, for negative affect as measured using the PANAS, two further models included a significant interaction with interactions between adventurous play and household income (*b* = 0.00 [− 0.01, 0.00], *p* = 0.012) and between unadventurous play and household income (*b* = 0.00 [0.00, 0.01], *p* = 0.012).

To explore each of these interactions we divided the sample into lower and higher household income groups using the social grade variable and calculated correlations. The results show that, for low household income, there were statistically significant negative associations between SDQ internalising score and time spent playing both outdoors (*r* =  − 0.14* [− 0.21, − 0.07]) and adventurously (*r* =  − 0.15* [− 0.22, − 0.08]) but neither of these correlations were significant for high household income (outdoors: *r* =  − 0.01 [− 0.07, 0.05]; adventurously: (0.01 [− 0.05, 0.07]). The relationship between positive affect and time spent playing adventurously was stronger for low household income (*r* = 0.25* [0.19, 0.32]) than for high household income (*r* = 0.11* [0.05, 0.17]), although significant for both groups. In contrast, for high household income, negative affect was negatively associated with time spent playing unadventurously (*r* =  − 0.14* [− 0.20, − 0.08]), and positively associated with time spent playing adventurously (0.09* [0.03, 0.16]), whereas these relationships were smaller and not statistically significant for low household income (unadventurously: *r* =  − 0.02 [− 0.09, 0.05]; adventurously: *r* =  − 0.03 [− 0.10, 0.04]).

Linear regressions controlling for demographic variables showed that the interactions between play and household income remained significant for the models predicting internalising problems and positive affect (see results file available here: https://beta.ukdataservice.ac.uk/datacatalogue/doi/?id=8793#!#0), but did not remain significant in either model predicting negative affect.

### Brief Discussion

The results supported our primary hypotheses that children who spent more time playing adventurously would have fewer internalising problems as well as more positive affect during the Covid-19 lockdown. We also hypothesised that adventurous play would be associated with less negative affect but, consistent with Study 1, this was not supported. A significant negative association was found, however, between unadventurous play and negative affect; children who typically spent more time playing unadventurously had less negative affect during the Covid-19 lockdown. For all associations, the effect sizes were small.

Complementing Study 1, we found interactions between play and household income indicating that adventurous play may be more closely related to internalising problems and positive affect for children from low-income families. Surprisingly, we also found an interaction between adventurous play and family income for negative affect, whereby children from a higher income family who spent more time playing adventurously had more negative affect, whereas no significant association was found for children from lower income families. This latter interaction did not hold after controlling for demographic characteristics and should therefore be interpreted with caution.

Overall, the pattern of results is largely consistent with Study 1 and the findings were robust after controlling for a broad range of socio-demographic factors. The results provide some indication of specificity because no significant association was found between adventurous play and externalising problems. Notably, similar patterns for outdoor play and adventurous play were found in Study 2, making it difficult to determine whether adventurous play specifically is driving these findings.

## Discussion

It has been proposed that to prevent anxiety and broader mental health problems, children need opportunities to play adventurously and take risks in their play. We therefore evaluated, across two studies, the hypothesis that more adventurous play is associated with fewer internalising problems. In both studies, there was support for this hypothesis. In Study 2, the correlation was stronger for children growing up in households with lower income. Although the effect sizes were notably small, the association between adventurous play and internalising problems was robust, holding after a wide range of demographic factors were accounted for, and when an alternative cutoff was used to define adventurous play. The small effect size is arguably to be expected given the broad range of factors that affect children’s mental health; any individual factor such as play experience would be expected to account for only a small proportion of variance. The variation in effect size due to household income may be due to corresponding differences in exposure to adventure via more structured activities. For example, activities such as scouts, martial arts or adventure camps all provide children with exposure to the feelings of uncertainty, arousal and coping that are proposed to underpin associations between adventurous play and anxiety. These opportunities may be less abundant for children from families with lower household income and therefore, adventurous play may have be more important for these children. The CPS measures unstructured play so it does not capture these additional activities, but this explanation would be in keeping with previous research focused on the benefits of adventure experiences (e.g. [34]).

We also hypothesised that children who typically spend more time playing adventurously may have coped better with the uncertainty of the Covid-19 pandemic and lockdown and may therefore have experienced more positive affect and less negative affect during this period. The results partially supported this hypothesis; across both studies a clear positive association between adventurous play and positive affect was found. Children who typically played more adventurously were happier during the first Covid-19 lockdown than those who did not. Again, although effect sizes were small they were robust across studies and held after controlling for a range of other demographic factors. In contrast, no direct associations between adventurous play and negative affect were found. Although there was some indication that children from higher income families may have experienced more negative affect during lockdown if they usually played more adventurously, this did not hold after controlling for other demographic factors. The effects for positive affect are of relevance to mental health given that low positive affect is a core feature of depression. Children with higher positive affect have lower symptoms of depression over time [35]. This finding therefore provides some initial support for the idea that adventurous play may help children to cope, or at least maintain positive affect, during difficult periods, even if it does not prevent negative affect.

An additional aim was to explore the specificity of any associations found between adventurous play and internalising problems. Theoretical models propose that whilst play offers broad benefits for children’s development, happiness and wellbeing, it is adventurous play specifically that provides learning opportunities which buffer against anxiety. Our findings broadly support this; no associations with externalising problems were found and unadventurous play was either not associated with internalising symptoms or was positively associated such that more unadventurous play was associated with more internalising symptoms. Notably, there is significant overlap between our adventurous play and outdoor play measures, as indicated by the high correlation, but results were less consistent across the two studies for outdoor play than for adventurous play.

From a theoretical perspective, the results provide some initial support for the predictions of a number of models [8, 9] but the data are cross-sectional and we cannot rule out the the direction of effect is that children with higher internalising problems play less adventurously. We also cannot conclude that adventurous play experience definitely helped children to cope during lockdown, without having a baseline, or pre-pandemic, measure of positive affect or a control group not exposed to the pandemic. Nevertheless, this research provides an important first step in evaluating theoretical claims and provides a foundation for future longitudinal and experimental work. For example, where it may be possible to manipulate adventurous play (i.e. through school-based interventions) and directly assess the impact on children’s mental health.

The research has a number of strengths including the use of two independent samples, one of which was large and nationally representative. There are some significant limitations. The primary limitation is the reliance on parent report of play and mental health. We are confident that the results are not due to shared method variance due to the specificity of the associations, but parent report offers only one perspective on both the child’s play and the child’s mental health. It is also important to acknowledge that parents were asked to report retrospectively about their child’s play and mental health due to the national lockdown as a result of Covid-19. It is not possible to evaluate the accuracy of this retrospective report but the measures were completed very soon after the start of the lockdown (a maximum of one month later) which means we can be reasonably confident that parents would have been able to report with reasonable accuracy [36]. Furthermore, due to the large sample, we relied on questionnaire measures but it may be beneficial for future work with more focused samples to use diary-based measures of play perhaps combined with technology to allow tracking of activity levels or geographical location. Finally, adventurous play is likely to offer a range of benefits to children, including an increased sense of autonomy, improved risk perception and greater physical activity, but these outcomes were not assessed given our focus on children’s mental health. Future research would benefit from taking a holistic view of children’s development and well-being to better capture the effects of this type of play.

To conclude, the results indicate that providing children with opportunities to play adventurously may have a small but positive effect on their mental health, particularly for children from low income families. Importantly, small effects can have substantial real world consequences due to their ability to accumulate over time and at scale [37]. This has been argued to be particularly important when the outcomes are consequential (e.g., mental health). For example, whilst universal free school breakfasts are related to a small increase of 0.09 standard deviations in math achievement, over a year duration the benefits of free school breakfast may equate to around 1.6 months of schooling per child [38]. Given that children enjoy playing in an adventurous way, interventions to support children’s access to and engagement in adventurous play are likely to be an appealing way to support children’s mental health.

## Summary

A number of theories indicate a role for adventurous or risky play in anxiety prevention. In this paper for the first time, the association between children’s time spent playing adventurously and their mental health is examined. More adventurous play was associated with lower internalising symptoms and more positive affect. The results suggest that giving children more opportunity to play in an adventurous way when they are in and out of school may offer benefits in terms of children’s mood and longer term mental health. To support children’s mental health, they need opportunity to play outdoors and adventurously, planning policy must consider children’s needs to ensure that every child, particularly those growing up in lower income families, has free access to safe space for adventurous outdoor play close to home. Longitudinal and experimental work is required to tease apart direction of effect in future research.

## Supplementary Information

Below is the link to the electronic supplementary material.

Supplementary file1 (DOCX 5489 kb)

## Data Availability

The surveys, data, analysis script and results files that support the findings of this paper are openly available via the UK Data Service: https://beta.ukdataservice.ac.uk/datacatalogue/doi/?id=8793#!#0.
