# Supplementary material for: Child’s Play: Examining the Association Between Time Spent Playing and Child Mental Health
Source: Child Psychiatry Hum Dev. 2022 May 14;54(6):1678–86. doi: 10.1007/s10578-022-01363-2 (PMC9106508; doi:10.1007/s10578-022-01363-2)
Supplement: Supplementary file 1 — Supplementary file1 (DOCX 5489 kb) [file 10578_2022_1363_MOESM1_ESM.docx]

**Supplementary Material**

**Contents**

1. Children’s Play Scale additional details
2. Study 1 additional detail for methods
   1. Screening for bot responses
   2. Missing data
   3. Distributions and outlier checks
3. Study 2 additional detail for methods
   1. Missing data
   2. Distributions and outlier checks
4. Replication of results section from BCPS paper (Dodd et al., 2021) using Study 1 data from Northern Ireland

**Section 1**

**Children’s Play Scale additional details**

The CPS has questions about play in seven places: at home or in other people’s homes; outside at home or at other people's homes (e.g. garden/yard/balcony); at a playground; in trees/forests/woodland/grassy spaces (not including the garden at home or other people's homes); in the street or public places close to home; outdoors near water (e.g. at the beach, in the sea, near a river, lake or cliffs); indoor play centres and pools (e.g. soft play, trampoline parks, swimming pools etc.). For ease, the ‘trees/forests/woodland/grassy spaces’ category will be referred to as ‘green space’, ‘street or public places’ will be referred to as ‘street’ and the final category will be referred to as ‘indoor play centres’.

Respondents were asked to report the frequency with which their child plays in each place and the length of time their child plays for in each place; Autumn/Winter and Spring/Summer were reported on separately. Frequency questions were answered on a seven-point scale (every day, 4 to 6 times a week, 2 to 3 times a week, once a week, 1 to 3 times a month, less than once a month, never), time questions were answered on a six-point scale (less than half an hour; half an hour to an hour; 1 to 2 hours; 2 to 3 hours; 3 to 4 hours; more than 4 hours). Frequencies were converted into approximate number of days within the six-month seasonal window (e.g. 4 to 6 times a week was converted into 5 times a week on average across 6 months of autumn/winter to give 130 days). Similarly, times were converted into approximate times per day that the child played in the place (e.g. 1 to 2 hours was converted into 90 minutes per day of play). Frequencies and times were multiplied to give the approximate number of minutes a child played in a specific place for within a 6-month season. These time estimations for each season were then summed to give an estimate of total time spent playing in each place within a year.

The adventurous play supplement was also conducted. This asked parents to rate how adventurously their child plays when they play at each place. This was rated on a five-point Likert scale ranging from 1 (very low levels of adventure) to 5 (maximum levels of adventure). Definitions of each point on the scale were provided.

**Section 2**

**Study 1 additional detail for methods**

a. bot screening process

We included two questions to screen for bots and participants not paying attention. At the beginning the survey stated:

We have had problems in the past with bots completing our surveys so this survey includes some questions to prevent this. They may seem like odd questions!

Could you type the surname of the US president backwards.

This was followed by a text box.

At the end of survey we also asked the following:

One final question to check that you're a real person paying attention (sorry!). What has this survey been about?

This was followed by a text box.

Through this process we filtered out 346 responses to give the 427 genuine participants included in Study 1. This proportion of bot responses aligns with research on this subject (Griffin et al., 2021)

Reference

Griffin, M., Martino, R.J., LoSchiavo, C., Comer-Carruthers, C., Krause, K.D., Stults, C.B., & Halkitis, P.N. (2021). Ensuring survey research data integrity in the era of internet bots. Qual Quant. https://doi.org/10.1007/s11135-021-01252-1

b. Missing data

Some demographic data were missing due to participants choosing not to respond to certain items (see Table S1). This missing data means that the total number of participants included in the models with demographic factors as predictors is very slightly reduced from the full sample.

For the CPS, due to an error in the programming of the survey, three items (time spent playing during the spring/summer for indoor play centres, street play and play near water) were missing for 157 participants. In addition, there was missing data on item 6 of the PANAS (n=80), item 2 of the K-6 (n=1) and item 6 of the K-6 (n=2). These data were imputed using the mice package in R to create 5 imputed datasets. All other available raw variables were used as predictors and the pmm imputation method was used. The multiple imputation ran without errors with the exception of variables identified as constants, which were removed as predictors automatically. Once the imputation was complete there were no missing data for variables capturing time spent playing across place and therefore no missing data for total time spent playing per year or for total time spent playing outside per year. There was no missing data for time spent playing adventurously. The results reported are for pooled findings unless otherwise specified.

c. Distribution and Outlier Checks

All of the time spent playing variables for each place (e.g. at home, outside at home etc.) were Winsorized such that any values lower than the 5th percentile or higher than the 95th percentile were given the value of the 5th and 95th percentile respectively. These Winsorized variables were used to create the variables for analysis. All of these resulting variables were positively skewed and were square-root transformed. No outliers were identified so no further Winsorizing was required. The SDQ internalising and externalising scores, the PANAS negative affect score, and the Kessler-6 score were all positively skewed and were also square-root transformed. These transformed variables and the PANAS positive affect score were Winsorized except for SDQ internalising and Kessler-6 which did not have outliers.

**Section 3**

**Study 2 additional detail for methods**

Missing data

For the CPS, there were no missing data for time spent playing outside per year. For the questionnaire items on level of adventurous play, participants were only asked to rate how adventurously their child played for those places where they had previously stated that their children played. Any items they did not see were coded as missing. The adventurous play supplement included a ‘don’t know’ response in Study 2, which was also coded as missing. This means that for individual places there were missing data for level of adventurous play as follows ( ‘don’t know’ responses in brackets): Home = 47 (29); Outside at home = 66 (22); Playground = 80 (26); Green space = 154 (40); Near water = 428 (46); Indoor play centre = 164 (36); Street = 727 (37). For hours spent playing adventurously 131 participants (7%) had missing data due to a ‘don’t know’ response to at least one of the adventurous play rating questions.

Due to a programming error, the PANAS item *mad* was replaced with the item *lonely* for a substantial number of participants. There was also missing data on the K6 as participants had the right to skip this measure given the nature of the questions. These data were imputed using the mice package in R to create 5 imputed datasets. All other available raw variables without missing data were used as predictors and the pmm imputation method was used. The multiple imputation ran without errors with the exception of variables identified as constants, which were removed as predictors automatically. Once the imputation was complete there were no missing data for mental health variables. The results reported are pooled results unless otherwise specified.

Some demographic data were missing due to participants choosing not to respond to certain items. This missing data means that the total number of participants included in the models with demographic factors as predictors is slightly reduced from the full sample.

Distribution and Outlier Checks

All of the time spent playing variables for each place (e.g. at home, outside at home etc.) were Winsorized such that any values lower than the 5th percentile or higher than the 95th percentile were given the value of the 5th and 95th percentile respectively. These Winsorized variables were used to create the variables for analysis. All of these resulting variables were positively skewed and were square-root transformed. For time spent playing adventurously and time spent playing outdoors, no outliers were identified so no further Winsorizing was required. For time spent playing unadventurously, outliers were identified and the variable was Winsorized. The SDQ internalising and externalising scores, the PANAS negative affect score, and the Kessler-6 score were all positively skewed and were also square-root transformed. These transformed variables and the PANAS positive affect score were Winsorized except for SDQ internalising and Kessler-6 which did not have outliers.

**Section 4**

**Replication of results section from BCPS paper (Dodd et al., 2021) using Study 1 data from Northern Ireland**

In addition to the methods detailed in the main body of the paper, the following measures were collected.

CPS

To mirror the analyses reported in the British Children’s Play Survey (BCPS), time spent playing unadventurously was not included in these analyses and instead we included a total time spent playing variable. This variable was calculated by summing the estimated time spent playing in each of the seven locations.

The Risk Engagement and Protection Survey (REPS)

The REPS assessed parent and caregiver attitudes and views around protecting children from injury and allowing them to engage in risks. Respondents are asked to indicate the extent to which they agree with 14 statements such as “Benefits of physical activity for my child outweigh the risk of experiencing minor injuries”. We calculated two subscale scores following Olsen, Ishikawa (38): Protection from Physical Injury (PfI) and Engagement with Risk (EwR). In keeping with Jelleyman, McPhee (39), these scores were calculated using 12 out of the 14 items. The subscales yield scores ranging from 6 to 42 with higher scores indicating greater engagement with risk and protection from injury. The internal consistency was good for PfI (alpha = .87) and acceptable for EwR (alpha = .69).

The Tolerance of Risk in Play Scale (TRiPS)

The TRiPS (40) evaluates adult tolerance of risk during children’s play. Respondents answer ‘yes’ or ‘no’ to 32 items that vary in the how ‘easy’ they are to endorse. Following the scoring used in Jelleyman, McPhee (39) a ‘no’ response is scored 0 and a ‘yes’ response receives a score from 1-12, with the weighting determined by how acceptable the level of risk referred to in the item is. These scores were determined via a Rasch analysis conducted within the original validation study for the TRiPS (40). For example, ‘Would you allow your child to play chase with other children?’ is a relatively high acceptability item so would receive a score of 2 and ‘Would you let your child use a hammer and nail unsupervised?’ is an example of a relatively low acceptability item and would receive a score of 8. After all items are completed, a risk tolerance score is calculated by summing the scores for all items, yielding scores that range from 0-184, with higher scores indicating greater risk tolerance. For descriptive purposes, in line with previous work (39), parents were categorised into one of four categories based on their score of risk tolerance: risk averse (0-61), somewhat risk averse (62-95), somewhat risk tolerant (96-122) and risk tolerant (123-184). These categories were determined in a previous study using representative data from New Zealand by dividing scores into quartiles (39).

Age allowed out alone

Parents/carers were asked what age they were when they were allowed to play out alone in their local neighbourhood and what age they did/would allow their child to play out alone in their local neighbourhood.

Distribution and Outlier Checks

The Engagement with Risk scale of the REPS had some extreme low values, and outliers were identified for the age allowed out alone question, both for child age and respondent age. These variables were therefore also Winsorized as described above.

**Table S1.** Demographic characteristics of full sample

| **Characteristic** | ***N* (%)** |
| --- | --- |
| Parent sex | 427 |
| Male | 27 (6%) |
| Female | 400 (94%) |
|  |  |
| Child sex | 427 |
| Male | 233 (55%) |
| Female | 194 (45%) |
|  |  |
| Parent age | 427 |
| 18-24 | 1 (<1%) |
| 25-34 | 135 (32%) |
| 35-44 | 240 (56%) |
| 45-54 | 48 (11%) |
| 55+ | 3 (<1%) |
|  |  |
| Relationship to child | 427 |
| Mother | 396 (93%) |
| Father | 26 (6%) |
| Stepparent | 3 (<1%) |
| Grandparent | 1 (<1%) |
| Others | 1 (<1%) |
|  |  |
| Child birth-order | 427 |
| First-born | 314 (74%) |
| Second-born | 67 (16%) |
| Third or more | 42 (10%) |
| Other | 4 (<1%) |
|  |  |
| Parent ethnicity | 427 |
| White British | 239 (56%) |
| White Irish  White (other background) | 163 (38%)  11 (3%) |
| Black | 0 (0%) |
| Asian | 1 (<1%) |
| Multi-ethnic | 7 (2%) |
| Other/Prefer not to say | 6 (1%) |
|  |  |
| Employment status | 427 |
| Working full-time | 200 (47%) |
| Working part-time | 140 (33%) |
| Student | 3 (1%) |
| Retired | 0 (0%) |
| Unemployed or not working | 72 (17%) |
| Other | 12 (3%) |
|  |  |
| Parent education level | 427 |
| Low | 112 (26%) |
| Medium | 92 (22%) |
| High | 213 (50%) |
| Don’t know/prefer not to say | 10 (2%) |
|  |  |
| Marital status | 427 |
| Married, living as married, civil partnership | 347 (81%) |
| Separated, divorced or widowed | 24 (6%) |
| Never married | 42 (10%) |
| Prefer not to say | 14 (3%) |
|  |  |
| Caregiving responsibility | 427 |
| Primary/shared  Other | 427 (100%) |
|  |  |
| Child disability | 427 |
| Yes | 67 (16%) |
| No | 349 (82%) |
| Prefer not to say | 3 (<1%) |
| Don’t know | 8 (2%) |

***Supplementary Results***

The following results use the data collected from parents and caregivers in Northern Ireland. The research questions mirror those addressed in the publication of the British Children’s Play Survey.

Research question 1: Where do children aged 5-11 years living in Northern Ireland spend time playing? Does the amount of time that they spend playing in each place vary significantly?

To address research question 1, three variables were used from the CPS: total time spent playing across the year, total time spent playing outside across the year, and total amount of time spent playing adventurously across the year. These three play variables were highly correlated (r > .59) as would be expected given that there is overlap in the items that create each variable.

On average, children were estimated to spend 1618 h (*SD* = 709 h) playing per year (4.43 hours per day), with 952 h (*SD* = 564 h), or 59%, spent playing outside, and 177 h (*SD* = 181 h), or 11%, playing in nature. Figure 1 shows the average amount of time that children were reported to spend playing at each of the locations included in the CPS, across a year. To examine whether the average total time children spent playing varied significantly across place, we conducted a series of linear models. Due to the data structure it was not possible to pool the analyses across the imputed datasets but the pattern of results was consistent when each imputed dataset (and the raw dataset) was analysed separately. Significant differences were found between each place and each other place (*ps*  < .001), with the exception of play in the street or local area close to their home, play in green spaces, and play at playgrounds, which were comparable, and indoor play and play near water, which were also comparable. Children spent the most time playing at home or at other people’s homes and the least time playing near water and at indoor play facilities, including swimming pools, trampoline parks and soft play. Away from home, children on average spent more time playing in the street or local area close to their house than in any other place.


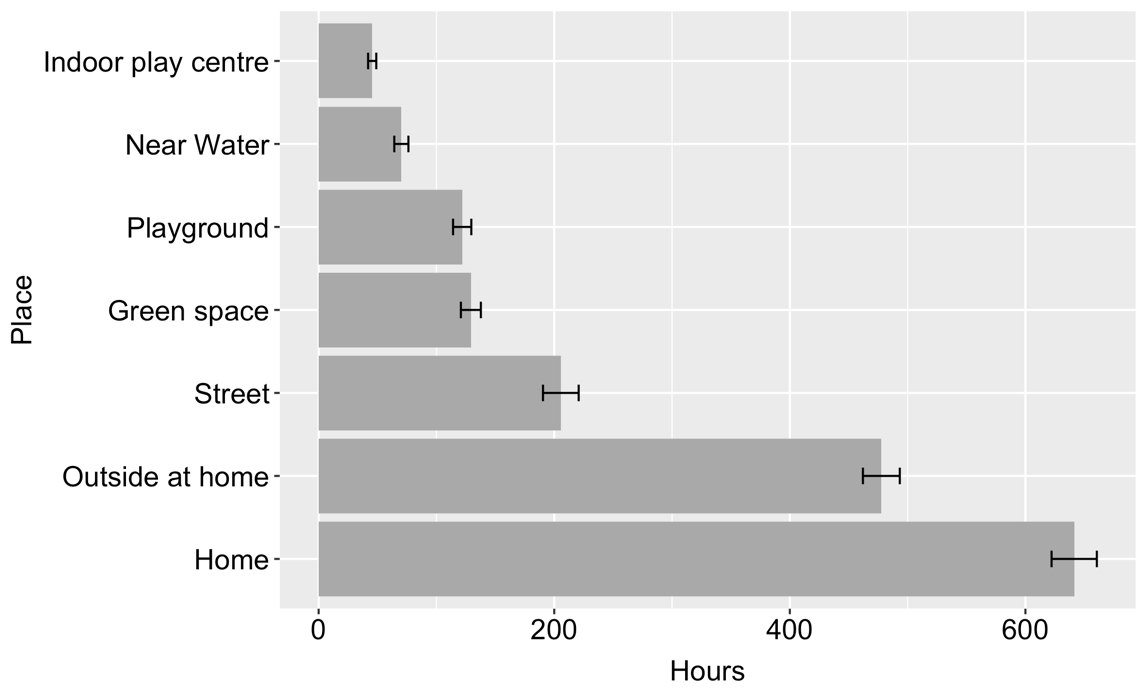


Research question 2: How adventurously do children living in Northern Ireland play? Does this vary by play location?

Figure 2 shows the average level of adventure parents perceived children to play at in each location. Linear models were used to examine the extent to which place predicted adventure level. These analyses showed that place was a significant predictor of adventure level. The adventure level of each place differed significantly (*ps*  < .002) from the adventure level of each other place with the exception of green space, indoor play centres and playgrounds, which were comparable, and street play and home play, which were also comparable. The highest levels of adventure happening during play in green spaces and at indoor play centres (including soft play, trampoline parks, swimming pools). The highest levels of adventurous play happened away from children’s homes and the areas immediately surrounding their homes.


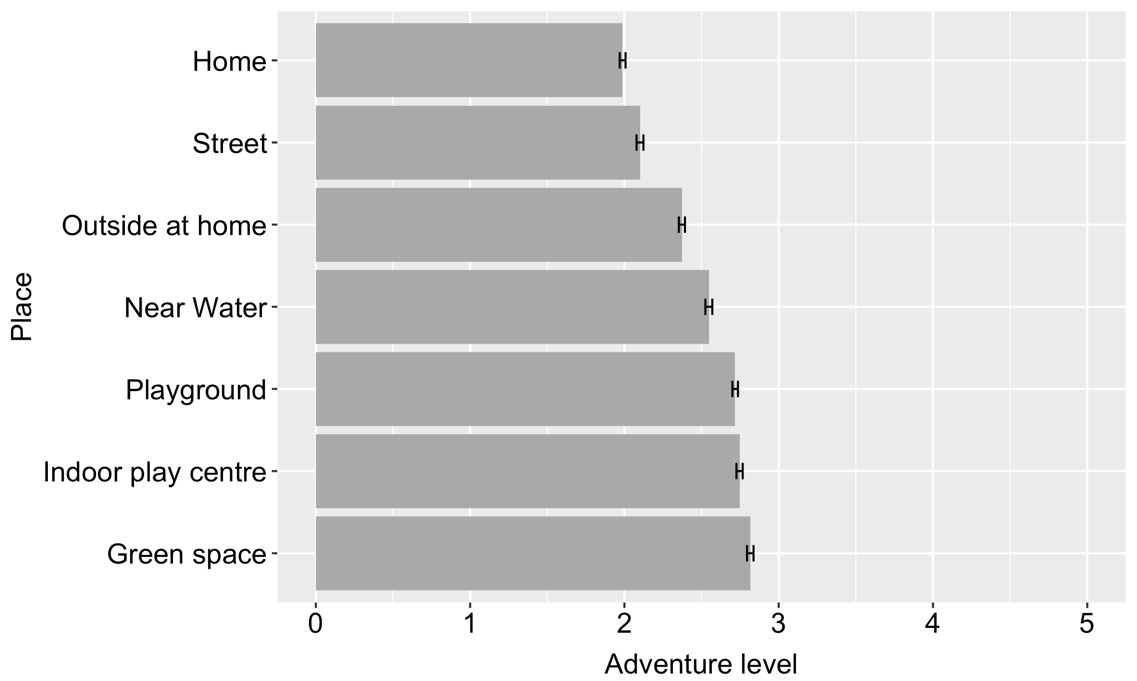


Research question 3: What age are children living in Northern Ireland allowed out in their neighbourhood alone?

Parents reported that they were allowed out alone on average at 8.23 years (SD = 2.21). They reported that their children were allowed out alone at age 9.69 years (SD = 2.46). A paired samples t-test showed that these values were significantly different, t (426) = 13.07, *p* < .001.

Research question 4: To what extent are, socio-demographic factors and parent attitudes to risk and protection associated with children’s total time spent playing, time spent playing outdoors and time spent playing adventurously?

Socio-Demographic Factors

Child sex, child age, child disability, child ethnicity, child birth-order, respondent employment status, marital status, respondent age and respondent education level were examined as predictors of children’s play. All were categorical variables except for child’s age and respondent age (which were mean centred). Given that some variables had low numbers within some subcategories, we collapsed across subcategories for the following variables: ethnicity (collapsed into White British, White Irish and other); birth order (collapsed into first born/not first born); education level (collapsed into low/medium/high, using the categorisation system used in previous research); employment (collapsed into three categories employed full-time/employed part-time/unemployed and other; this final category included students, retired, unemployed, not working and other). Three generalised linear models were used to evaluate whether and how these socio-demographic factors were associated with children’s play. The results are shown in Table 2.

Using an alpha level of *p* <.05 to indicate significance, total hours spent playing was predicted by child age and parent level of education only, with children of highly educated parents playing less than children of parents with low levels of education. Children played less as they got older. Outdoor play was only significantly predicted by parent education level, with parents of highly educated parents playing outdoors less than children whose parents had low levels of education. Adventurous play was only predicted by birth order, with first born children spending more time playing adventurously than children who were not first born. The proportion of variance accounted for by the sociodemographic predictors ranged from 3% for adventurous play to 6% for hours spent playing.

Table 4. Socio-demographic predictors of hours spent playing, hours spent playing outside and hours spent playing adventurously per year. Results are pooled across imputations.

|  | **Hours playing p/yr** | | | **Hours playing outside p/yr** | | | **Hours playing adventurously p/yr** | | |
| --- | --- | --- | --- | --- | --- | --- | --- | --- | --- |
| *Predictors* | *Estimates* | *CI* | *p* | *Estimates* | *CI* | *p* | *Estimates* | *CI* | *p* |
| (Intercept) | 39.68 | 36.4 – 42.96 | **<.001** | 30.12 | 26.76 – 33.48 | **<.001** | 34.10 | 28.92 – 39.28 | **<.001** |
| Child age | -0.55 | -1.03 – -0.08 | **.023** | -0.09 | -0.58 – 0.4 | .719 | -0.41 | -1.17 – 0.35 | .288 |
| Child sex: Male | *Reference* |  |  | *Reference* |  |  | *Reference* |  |  |
| Child sex: Female | 1.50 | -0.32 – 3.32 | .106 | 1.40 | -0.47 – 3.27 | .143 | -0.20 | -3.09 – 2.7 | .894 |
| Child disability: No | *Reference* |  |  | *Reference* |  |  | *Reference* |  |  |
| Child disability: Yes | -1.61 | -4.17 – 0.94 | .216 | -0.20 | -2.83 – 2.43 | .882 | -1.36 | -5.44 – 2.72 | .514 |
| Ethnicity: White British | *Reference* |  |  | *Reference* |  |  | *Reference* |  |  |
| Ethnicity: White Irish | 0.45 | -1.45 – 2.35 | .641 | 0.22 | -1.74 – 2.18 | .824 | -0.74 | -3.76 – 2.27 | .628 |
| Ethnicity: Any other background | -2.76 | -6.76 – 1.24 | .176 | -1.65 | -5.83 – 2.53 | .438 | -2.74 | -9.13 – 3.66 | .401 |
| Employment: Full time | *Reference* |  |  | *Reference* |  |  | *Reference* |  |  |
| Employment: Part time | -0.49 | -2.54 – 1.55 | .636 | -0.12 | -2.22 – 1.99 | .914 | 0.65 | -2.6 – 3.9 | .696 |
| Employment: Unemployed/other | -0.55 | -3.14 – 2.05 | .678 | -0.75 | -3.42 – 1.92 | .582 | -1.06 | -5.17 – 3.05 | .611 |
| Birth order: First born | *Reference* |  |  | *Reference* |  |  | *Reference* |  |  |
| Birth order: Not first born | -2.09 | -4.36 – 0.17 | .070 | -1.13 | -3.47 – 1.20 | .340 | -3.73 | -7.31 – -0.15 | **.041** |
| Parent marital status: Married/civil partnership/living as married | *Reference* |  |  | *Reference* |  |  | *Reference* |  |  |
| Parent marital status: Any other response | 1.93 | -0.47 – 4.33 | .115 | 1.22 | -1.23 – 3.68 | .328 | 1.25 | -2.53 – 5.02 | .517 |
| Parent age | -0.05 | -0.22 – 0.13 | .609 | -0.10 | -0.28 – 0.09 | .298 | -0.03 | -0.32 – 0.25 | .827 |
| Parent education: Low | *Reference* |  |  | *Reference* |  |  | *Reference* |  |  |
| Parent education: Med | -1.58 | -4.28 – 1.11 | .249 | -1.57 | -4.36 – 1.21 | .267 | -1.45 | -5.73 – 2.84 | .507 |
| Parent education: High | -2.88 | -5.21 – -0.54 | **.016** | -3.20 | -5.61 – -0.78 | **.010** | -1.22 | -4.93 – 2.49 | .518 |
| R^2^ / Adjusted R^2^ | .064 / .036 | | | .039 / .009 | | | .031 / .001 | | |

Parent attitudes to risk

Figure S1 shows the proportion of parents whose total scores on the TRiPS fall into each categories of risk tolerance. This shows that the majority of parents were relatively risk averse.


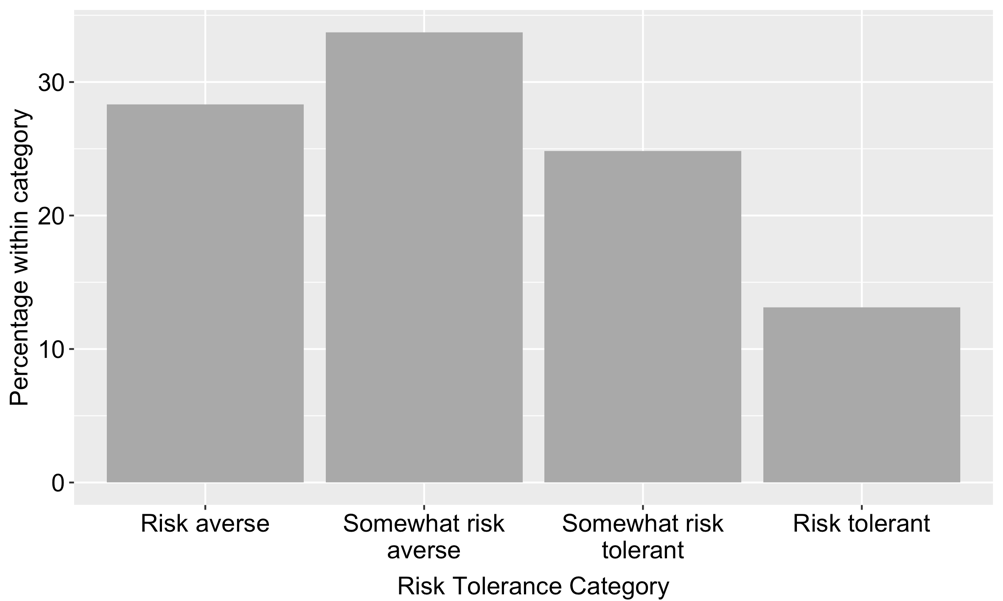


Linear models were conducted to examine whether parent attitudes to risk, as measured by the REPS and TriPS were associated with children’s time spent playing. Hours spent playing, hours spent playing outdoors and hours spent playing adventurously was significantly predicted by EWR scale scores, with children playing more when their parent had higher EWR scores. The amount of variance in play accounted for by parent attitudes was low, ranging from 2% for total time spent playing to 5% for adventurous play.

Table 5. Parent attitude towards risk predictors of hours spent playing, hours spent playing outside and hours spent playing adventurously per year.

|  | **Hours playing p/yr** | | | **Hours playing outside p/yr** | | | **Hours playing adventurously p/yr** | | |
| --- | --- | --- | --- | --- | --- | --- | --- | --- | --- |
| *Predictors* | *Estimates* | *CI* | *p* | *Estimates* | *CI* | *p* | *Estimates* | *CI* | *p* |
| (Intercept) | 39.16 | 38.28 – 40.03 | **<.001** | 29.39 | 28.5 – 30.29 | **<.001** | 32.66 | 31.31 – 34 | **<.001** |
| Engagement with risk | 1.23 | 0.26 – 2.2 | **.013** | 1.02 | 0.03 – 2.01 | **.043** | 2.12 | 0.62 – 3.61 | **.006** |
| Protection from injury | -0.24 | -1.22 – 0.73 | .624 | -0.36 | -1.35 – 0.63 | .470 | -0.56 | -2.06 – 0.94 | .462 |
| TriPs | 0.12 | -0.94 – 1.18 | .826 | 0.77 | -0.32 – 1.85 | .165 | 1.21 | -0.42 – 2.85 | .146 |
| R^2^ / Adjusted R^2^ | .022 / .015 | | | .032 / .025 | | | .047 / .040 | | |

Research question 5: To what extent are socio-demographic factors and parent attitudes to risk and protection associated with children’s independent mobility?

To examine the extent to which socio-demographic factors predict the age that children were allowed out alone, we conducted a linear model with the previously outlined range of sociodemographic predictors. No socio-demographic factors were significant predictors of the age that children were allowed out alone (ps > .05). Similarly, parent attitudes to risk did not significantly predict the age children were allowed out alone. The proportion of variance accounted for was small; 3% for socio-demographic factors and 2% for parent attitudes.

Table 6. Socio-demographic predictors of age children allowed out alone (independent mobility).

|  | **Age allowed out alone** | | |
| --- | --- | --- | --- |
| *Predictors* | *Estimates* | *CI* | *p* |
| (Intercept) | 9.90 | 9.02 – 10.78 | **<.001** |
| Child age | 0.16 | 0.03 – 0.29 | **.013** |
| Child sex: Male | *Reference* |  |  |
| Child sex: Female | -0.22 | -0.71 – 0.28 | 0.384 |
| Child disability: No | *Reference* |  |  |
| Child disability: Yes | 0.27 | -0.43 – 0.96 | .449 |
| Ethnicity: White British | *Reference* |  |  |
| Ethnicity: White Irish | 0.21 | -0.31 – 0.72 | .428 |
| Ethnicity: Any other background | 0.51 | -0.58 – 1.60 | .362 |
| Employment: Full time | *Reference* |  |  |
| Employment: Part time | -0.16 | -0.72 – 0.39 | .562 |
| Employment: Unemployed/other | 0.15 | -0.55 – 0.85 | .674 |
| Birth order: First born | *Reference* |  |  |
| Birth order: Not first born | -0.47 | -0.14 – 1.08 | .131 |
| Parent marital status: Married/civil partnership/living as married | *Reference* |  |  |
| Parent marital status: Any other response | 0.06 | -0.58 – 0.70 | .851 |
| Parent age | 0.02 | -0.02 – 0.07 | .315 |
| Parent education: Low | *Reference* |  |  |
| Parent education: Med | -0.24 | -0.97 – 0.50 | .528 |
| Parent education: High | -0.21 | -0.84 – 0.43 | .520 |
| R^2^ / Adjusted R^2^ | .031 / .001 | | |

Table 7. Parent attitude to risk as predictors of age children allowed out alone (independent mobility).

|  | **Age allowed out alone** | | |
| --- | --- | --- | --- |
| *Predictors* | *Estimates* | *CI* | *p* |
| (Intercept) | 9.69 | 9.46 – 9.92 | **<.001** |
| Engagement with risk | -0.12 | -0.38 – 0.13 | .346 |
| Protection from injury | 0.17 | -0.09 – 0.42 | .204 |
| TRiPs | -0.24 | -0.52 – 0.04 | .097 |
| R^2^ / Adjusted R^2^ | 0.028 / 0.021 | | |

***Supplementary Discussion***

The aim of these supplementary results was to provide an overview of play in Northern Ireland that supports comparisons with the recently published findings from British Children’s Play Survey. The data for the two studies was collected at approximately the same time and using almost identical measures but the sample from Northern Ireland were recruited via social media advertising whereas the BCPS sample were recruited via a market research organisation to be representative of the population of Great Britain (England, Scotland and Wales). The results indicate that children in Northern Ireland spend, on average, almost 4.5 hours a day playing. They spend more time playing at home than anywhere else. Away from homes and gardens, the most common place for children in this sample to play was in the street or neighbourhood near their home, followed by green spaces and playgrounds. Compared to the results of the BCPS, these childen in Northern Ireland spend substantially more time engaged in play (almost 1.5 hours a day more, on average) and notably more time playing on the street or in areas close to their home.

In relation to adventurous play, the results are almost identical to those for the BCPS; children play most adventurously in green spaces such as woodland, forests and open grassy areas, followed by indoor play centres such as soft play and trampoline parks and then at playgrounds. The average level of adventure was mild to moderate and comparable to the BCPS results.

Research question three examined the age that children are allowed out alone. Results showed that children in this sample from Northern Ireland were allowed out alone at an average of 9.7 years, whereas their parents reported being allowed out alone at 8.2 years, on average. This increase in the age that children are allowed out alone mirrors the finidngs of the BCPS where a difference of almost 2 years was found. It is noteworthy that the average age reported here is comparable to that found in the BCPS for Scotland (9.57 years). This stood out in the BCPS data because it was significantly different to the age that children in England and Wales were allowed out alone, which was at least a year later.

Across research questions four and five we examined whether demongraphic factors were predictors of children’s play and the age at which children were allowed out alone. In keeping with the BCPS results, demographic predictors accounted for only a very small proportion of the variance in any of the play or independent mobility variables, with the majority of the adjusted R-square values indicating that less than 1% of the variance was accounted for. The exception was for total hours spent playing where age and parent education level were significant predictors and the model accounted for 4% of the variance. Similarly, only a small proportion of variance was accounted for by parent attitudes to risk, with the largest effects found for the engagement with risk scale as a predictor of adventurous play. This scale captures parent’s positive attutudes about the benefits of risk taking for children. In contrast to the present findings, scores on this scale were associated with all play variables in the BCPS, rather than just adventurous play.
